# Multidimensionale Formate chirurgischer Anatomie in der studentischen Ausbildung der HNO-Heilkunde – ein Effektivitätsvergleich

**DOI:** 10.1007/s00106-024-01427-w

**Published:** 2024-02-07

**Authors:** Jan S. Grajek, Stefanie Rettschlag, Armin Schneider, Sebastian P. Schraven, Robert Mlynski, Sara M. van Bonn

**Affiliations:** 1 Klinik und Poliklinik für Hals-Nasen-Ohrenheilkunde, Kopf- und Halschirurgie „Otto Körner“, Universitätsmedizin Rostock, Doberaner Str. 137, 18057 Rostock, Deutschland; 2 Jade Hochschule, Fachbereich Ingenieurwissenschaften, Friedrich-Paffrath-Str. 101, 26389 Wilhelmshaven, Deutschland

**Keywords:** Virtual Reality, Computersimulation, Medizinstudierende, Digitale Gesundheit, Lehre, Virtual reality, Computer simulation, Medical students, Digital health, Teaching

## Abstract

**Hintergrund:**

Der technologische Wandel im Gesundheitswesen und die digitale Transformation der Lehre erfordern Neuerungen in der studentischen Lehre im Bereich der Medizin. Neue Technologien sind nötig, um die Bereitstellung und Nutzung diverser Lehr- und Lernformate von Bildungseinrichtungen unabhängig von Zeit und Ort zu ermöglichen. Ziel der Studie ist die Analyse der Effektivität verschiedener multidimensionaler Formate in der studentischen Lehre in der chirurgischen HNO-ärztlichen Anatomie.

**Material und Methoden:**

Während des Sommersemesters 2022 und des Wintersemesters 2022/2023 wurde das digitale Lehr- und Lernprogramm ausgeweitet, indem mit Studierenden unterschiedliche Visualisierungsformate (3-D-Brillen, Cardboards oder VR-Brille) im Rahmen eines hochstandardisierten Operationsverfahrens, der Cochleaimplantation, getestet wurden. Prä- und postinterventionell wurde in allen Gruppen eine Wissensstandserhebung und im Anschluss daran eine Evaluation durchgeführt.

**Ergebnisse:**

Von 183 Studierenden nahmen 91 Studierende vollständig an der Studie teil. Die postinterventionelle Wissensstandserhebung ergab unabhängig vom Visualisierungsformat einen signifikanten Anstieg der korrekten Antworten. Im direkten Vergleich antwortete die Operationssaal(OP)-Gruppe signifikant häufiger richtig als die Cardboard-Gruppe (*p* = 0,0424). Ein Großteil der Studierenden wünscht sich 3‑D-Lehre als festen Bestandteil im Lehrprogramm (87,9 %) und ein größeres Streamingangebot von Live-Operationen (93,4 %). Sie sehen die Anwendung der verschiedenen Technologien als sehr gute Ergänzung zur herkömmlichen chirurgischen Lehre (72,5 %), da bei guter Anschaulichkeit (89 %) die Merkfähigkeit (74,7 %) und Motivation (81,3 %) steigt.

**Schlussfolgerungen:**

Der Einsatz und die Nutzung neuer Visualisierungstechnologien im klinischen Alltag ist ein vielversprechender Ansatz zur Erweiterung der studentischen Ausbildung. Mobile, interaktive und personalisierte technische Formate sind an das Lernverhalten von Studierenden anpassbar. Nicht zuletzt wird durch den Einsatz neuer Medien die Lernmotivation beeinflusst. Eine Erweiterung digitaler Lehr- und Lernformate kann auf der Basis dieser Studie ausdrücklich empfohlen werden.

## Abstract (en)

**Background:**

Technological change in healthcare and the digital transformation of teaching require innovations in student teaching in medicine. New technologies are needed to enable the delivery and use of diverse teaching and learning formats by educational institutions independent of time and place. The aim of this study is to analyze the effectiveness of different multidimensional formats in student teaching in surgical ENT medical anatomy.

**Materials and methods:**

During the summer semester 2022 and winter semester 2022/2023, the digital teaching and learning program was expanded by testing different visualization formats (3D glasses, cardboards, or VR glasses) with students in the context of a highly standardized surgical procedure, namely cochlear implantation. A pre- and post-intervention knowledge assessment was carried out in all groups, followed by an evaluation.

**Results:**

Of 183 students, 91 students fully participated in the study. The post-intervention knowledge assessment showed a significant increase in correct answers regardless of visualization format. In a direct comparison, the operating room (OR) group answered correctly significantly more often than the cardboard group (*p* = 0.0424). The majority of students would like to see 3D teaching as an integral part of the teaching program (87.9%) and more streaming of live surgeries (93.4%). They see the use of the various technologies as a very good addition to conventional surgical teaching (72.5%), as good visualization (89%) increases retention (74.7%) and motivation (81.3%).

**Conclusion:**

Application and use of new visualization technologies in everyday clinical practice is a promising approach to expanding student training. Mobile, interactive, and personalized technical formats can be adapted to the learning behavior of students. Last but not least, the use of new media influences learning motivation. An expansion of digital teaching and learning formats can be expressly recommended on the basis of this study.

## „Digital natives“ und „virtual reality“

Der technologische Wandel im Gesundheitswesen und die digitale Transformation in der Lehre erfordern ein Überdenken und eine Überarbeitung von Bildungsreformen und -methoden. Der Stellenwert des Themas „digitale Kompetenzen im Studium“ rückt immer mehr in den Mittelpunkt. Nicht zuletzt die COVID-19-Pandemie hat gezeigt, dass die Entwicklungen digitaler Lehr- und Lernformate in verschiedenen medizinischen Bereichen hochrelevant sind. Es gilt, Studierende auf zukünftige Eventualitäten im beruflichen Alltag vorzubereiten, da der Gebrauch von digitalen Daten („data literacy“) von einigen Autoren bereits zu den entscheidenden und notwendigen Fähigkeiten im 21. Jahrhundert gezählt wird [1, 2]. Die Studierenden, als „digital natives“, sind in einer digitalen Welt mit verschiedenen Technologien aufgewachsen [3]. Von Kindheit an sind diese mit digitalen Technologien vertraut, und diese werden auch als selbstverständlich im Leben angesehen. Typischerweise bezieht sich der Begriff auf diejenigen, die in den letzten Jahrzehnten des 20. Jahrhunderts und zu Beginn des 21. Jahrhunderts geboren wurden. Es ist allerdings wichtig zu beachten, dass der Begriff „digital natives“ keine homogene Gruppe beschreibt, da die Erfahrungen und das Maß an technologischer Kompetenz innerhalb dieser Generation variieren können. Kuhn et al. beschreiben, dass trotz des Aufwachsens als „digital natives“ die Nutzung digitaler Technologien nicht unbedingt einen systematisierten Gebrauch im medizinischen Alltag bedinge [4]. Loda et al. vermerken jedoch auch positive Zusammenhänge diesbezüglich [1, 5]. Heutige Studierende sind Multitasking gewöhnt und erwarten somit auch, dass ihnen die Bereitstellung und Nutzung verschiedener Lehr- und Lernformate von Bildungseinrichtungen unabhängig von Zeit und Ort ermöglicht wird [6].

Im Medizinstudium sind klinische Unterrichtseinheiten eine zentrale Methode zur Vermittlung praktischer Fertigkeiten [1]. Im Rahmen der COVID-19-Panendemie wurden bereits zahlreiche innovative Lösungen zur Aufrechterhaltung und Sicherung der studentischen Ausbildung als Alternative zum Präsenzunterricht geschaffen (beispielsweise E‑Learning, Live-Online-Streaming von Vorlesungen oder Operationen). Diese haben sich bewährt und zeigten zumeist eine gute Akzeptanz, eine hohe Motivation und Nutzungsbereitschaft [7–10]. Es gilt, die universitäre Lehre flexibler zu gestalten, denn verkürzte Liegezeiten von Patienten, eine hohe Arbeitsbelastung in der Patientenversorgung, steigende Studierendenzahlen und wechselnde Dozierende erschweren einen strukturierten und qualitativ hochwertigen Unterricht [1]. Ziel ist es, Wissen einheitlich und gezielt übermitteln zu können, hierbei aber neue innovative Lehr- und Lernmethoden nicht außer Acht zu lassen. Doch inwiefern kann die Anwendung neuartiger Technologien sich als fester Bestandteil im Gesundheitswesen und der medizinischen studentischen Ausbildung etablieren oder sind digitale Lehr- und Lernformate nur Forschungsprojekte ohne Zukunftsaussichten?

„Virtual reality“ (VR) hat in der Medizin bereits einen großen Anklang und eine Vielzahl von Anwendungen gefunden. Durch immersive und interaktive Erfahrungen wird sie zunehmend als wirksames Werkzeug zur Verbesserung von Diagnostik und Behandlung, aber auch zur Weitervermittlung von Lehrinhalten eingesetzt. Im Rahmen der studentischen Ausbildung erschafft die virtuelle Realität eine neue und alternative, digitalisierte Welt, indem sie das Lernen auf eine interaktive Ebene hebt und es so u. a. ermöglicht, virtuelle Strukturen räumlich zu betrachten [11]. Hierfür verwendete VR-Brillen können komplexe 3‑D-Welten darstellen, aber auch als 3‑D-Visualisierungs-Tool und beispielsweise zur Übertragung von komplexen Operationen, dienen. Dies kann das Verständnis für die menschliche Anatomie im Sinne einer richtigen Einschätzung von Verhältnissen dreidimensionaler Strukturen vorteilhaft sein [12]. Zudem wird angenommen, dass durch den Einsatz neuartiger Technologien die Lernmotivation und -erfahrung steigt [7, 13, 14].

Ziel dieser Studie ist es, die Analyse der Effektivität verschiedener multidimensionaler Formate in der studentischen Lehre im Bereich der chirurgischen HNO-ärztlichen Anatomie zu analysieren. Hierfür erfolgte die Beurteilung bzw. der Vergleich von Akzeptanz und subjektivem Nutzen durch die Studierenden, die Untersuchung von Qualitätsmerkmalen solch neuartiger Lehrmethoden und die Überprüfung der Effektivität anhand von Wissensstandserhebungen und Effektivitätsanalysen.

## Methodik

### Studiendesign

Während des Sommersemesters 2022 und des Wintersemesters 2022/2023 wurde das digitale Lehr- und Lernprogramm an der medizinischen Fakultät einer Universitätsmedizin erweitert. Diese Studie wurde in einem prospektiven Design als Single-Center-Studie durchgeführt. Während regulärer Blockpraktika wurden die Studierenden, nach vorheriger Ankündigung und Vorstellung der Studie, zufällig 3 verschiedenen Gruppen zugewiesen: 3‑D-Gruppe, Cardboard-Gruppe oder VR-Brillen-Gruppe.

Bei der Cochleaimplantation, einem hochgradig standardisierten Eingriff, erfolgen die Präparationen anhand speziell definierter chirurgisch-anatomischer Landmarken an Patienten mit anatomisch-topografisch gesunden Felsenbeinen. Aufgrund von wiederkehrenden anatomischen Bezugspunkten eignet sich dieser Eingriff besonders gut für die Wissensstandserhebungen. Vor Beginn der Operation (Op.) gaben die betroffenen Patienten ihr schriftliches Einverständnis für die anonymisierte Übertragung von Op.-Videos zu Ausbildungszwecken.

### 3-D-Gruppe

Die 3‑D-Gruppe verfolgte die mikrochirurgische Op. direkt im Operationssaal (OP). Wie bereits in vorangegangenen Arbeiten der Autoren beschrieben, wurde innerhalb des Saals die Op. mit einem volldigitalen Op.-Mikroskop (ARRISCOPE Evo2 ENT, Fa. MSI, München, Deutschland) mit der Fähigkeit, ein 2‑D- und ein 3‑D-Bild zu übertragen, demonstriert. Operateur und Studierende betrachteten den exakt identischen Bildausschnitt des Op.-Felds. Während der Operateur die Sicht durch ein digitales Binokular hat, wurden den Studierenden die Live-Bilder auf 65-Zoll-3-D-Displays (LG 65EF9509, Fa. LG Electronics, Seoul, Republik Korea) präsentiert. Die Displays waren so positioniert, dass alle Studierenden einen direkten Blick, ohne Blickwinkelverzerrungen, auf den Bildschirm hatten. Alle Teilnehmer sahen die chirurgischen Eingriffe in 3‑D mit 4‑K-Qualität durch passive, polarisierte 3‑D-Brillen (Fa. Schleiter & Jauernig, Hamburg, Deutschland) in audiovisueller Echtzeit [7, 10].

### Cardboard- und VR-Brillen-Gruppe

Die Cardboard- und VR-Brillen-Gruppe verfolgten die zuvor aufgezeichnete mikrochirurgische Op. in High-Definition(HD)-Qualität. Im OP wurde der Eingriff mit einem volldigitalen 3‑D-Op.-Mikroskop (ARRISCOPE Evo2 ENT, MSI, München, Deutschland) aufgezeichnet. Der Chirurg war mit einem digitalen drahtlosen Taschensender (GLXD1, Fa. Shure, Niles, USA) ausgestattet, der mit einem Rackmount-Empfänger (GLXD4R, Fa. Shure, Niles, USA) verbunden war. Das Mikrofon war empfindlich genug, um die intraoperativen Diskussionen und Entscheidungsfindungen des gesamten Teams an die Studierenden zu übertragen. Die Audiospur des Rackmount-Empfängers sowie die Videospur des Op.-Mikroskops wurden mit einem Broadcast-Switcher (ATEM Mini, Fa. Blackmagic Design Pty Ltd, Victoria, Australien) verbunden, der an ein Konferenzraumsystem (Avaya Scopia XT7100, Fa. Avaya, Santa Clara, CA, USA) angeschlossen war. Tonspur und Videomaterial wurden über eine LAN-Schnittstelle („local area network“) an einen Mediendienstleister (Fa. Pripares, München, Deutschland) übertragen. Die Daten wurden vom Dienstleister umgewandelt und anschließend in 4‑K-HD-Qualität über einen eigenen Kanal im 3‑D-Format („side-by-side“) auf YouTube (Fa. Google LLC, Mountain View, USA) online gestellt [7, 10]. Die Studierenden der Cardboard- und VR-Brillen-Gruppe konnten die zuvor aufgezeichnete Op. in selbstausgewählter Geschwindigkeit sehen und hatten die Möglichkeit, das Video zu unterbrechen, um Abschnitte zu wiederholen.

Der Cardboard-Gruppe wurde ein faltbares Cardboard-VR-Headset (Fa. Silicon Foldable VR, Guangzhou, China) ausgeteilt, welches aus einem Plastikrahmen mit einem Smartphone-Einschub, der vor 2 optischen konvexen Linsen eingebracht ist, besteht. Das Smartphone fungiert als Bildschirm mit Bewegungssensoren (Gyroskope und Beschleunigungssensoren). Eine Smartphone-App erkennt die 3‑D-Quelle aus dem OP-Stream und stellt diese als Side-by-Side-Bild dar. Der Benutzer schaut durch die Linsen und bekommt ein vergrößertes Bild für jedes Auge separat angezeigt. Durch die Darstellung der 2 leicht divergierenden Bilder aus der mikroskopischen Aufnahme entsteht eine stereoskopische Wahrnehmung der Umgebung und damit ein 3‑D-Bild.

Die Studierenden der VR-Brillen-Gruppe erhielten eine Brille während der Lehreinheit (Pico G2 4K, Fa. ByteDance, Peking, China). Das Funktionsprinzip der VR-Brille ist mit dem des Cardboards vergleichbar, wobei Bildschirm, Bewegungssensoren sowie die Audioquellen in der Brille fest verbaut sind und eine freihändige Bedienung sowie Interaktion mit der virtuellen Umgebung mit einer Fernbedienung möglich ist. Die VR-Brille wird in dieser Studie nicht im eigentlichen VR-Sinn, sondern als 3‑D-Visualisierungs-Tool verwendet.

### Wissensstandserhebung

Zur Ermittlung des Wissensstands mussten alle Studierenden vor der Teilnahme an der Studie (präinterventionell) 11 anatomische Strukturen an Op.-Bildern mit Multiple-Choice-Fragen richtig zuordnen. Die Op.-Bilder wurden auf der Online-Plattform ILIAS (ILIAS Open Source E‑learning e. V., Köln, Deutschland) bereitgestellt. Das Open-Source-Produkt ILIAS ist ein frei verfügbares Learning-Management-System für die internetbasierte Bereitstellung von Lehr- und Lernmaterialien und ist sowohl im universitären Bereich wie auch von Unternehmen etabliert. Nach Ermittlung des Wissensstands nahmen die Studierenden an der Op. mit dem zuvor jeweils zufällig zugeteilten Tool teil. Anschließend erfolgte nach Abschluss der Op. (postinterventionell) die Wissensstandskontrolle durch erneute Zuordnung der 11 anatomischen Strukturen an den gleichen Op.-Bildern wie vor der Op. Zu den zu kennzeichnenden anatomischen Strukturen gehörten: Mastoidzellen, horizontaler Bogengang, Sinus sigmoideus, Dura zur mittleren und hinteren Schädelgrube, Chorda-Fazialis-Winkel, Verlauf des N. facialis, Chorda tympani, rundes Fenster, Amboss, Promontorium und Eminentia pyramidalis.

### Evaluation

Nach Abschluss der auf dem jeweiligen Tool gezeigten Op. wurden die Studierenden gebeten, einen Evaluationsbogen auszufüllen, der ebenso auf ILIAS bereitgestellt wurde (Tab. 1). Die 11 Fragen waren auf einer 5‑Punkte-Likert-Skala zu beantworten (1: „trifft zu“, 2: „trifft eher zu“, 3: „trifft teilweise zu“, 4: „trifft eher nicht zu“, 5: „trifft nicht zu“ und 6: „keine Antwort möglich“). Die Überprüfung der Qualitätsmerkmale (Video, Audio, Verbindungstabilität zum Stream, Verständlichkeit der intraoperativen Prozesse und Interaktionsmöglichkeit mit dem Operateur) erfolgte durch eine Ratingskala mit Abstufungen von sehr gut (1) bis mangelhaft (6).

**Table 1:** 

	Evaluationsbogen
1	Das OP-Streaming brachte mir einen großen Wissenszugewinn
2	Die angewandte Lernmethode ist anschaulich
3	Gezeigte Strukturen konnte ich mir durch die angewandte Methode besser merken
4	Durch das OP-Streaming (mit Tool) ist mein Interesse am Fachgebiet der HNO-Heilkunde gewachsen
5	Die eingesetzte Lernmethode motiviert mich und macht Spaß
6	OP-Streaming sollte fester Bestandteil der Lehre sein
7	Ich würde gerne mehr Operationen mit dem angewandten Tool sehen
8	Das OP-Streaming bietet eine gute Alternative zur herkömmlichen Op.-Lehre an
9	Das Angebot an OP-Streaming an der UMR sollte vermehrt erhöht werden
10	Besonders für distanzorientiertes Lernen eignet sich das OP-Streaming mit dem durchgeführten Tool besonders gut
11	Ich wäre bereit, für das OP-Streaming mit dem angewandten Tool Geld zu bezahlen
12	Qualität der Videoübertragung
13	Qualität der Audioübertragung
14	Verbindungsstabilität zum Stream
15	Verständlichkeit der intraoperativen Prozesse
16	Interaktionsmöglichkeiten mit dem Operateur

*OP* Operationssaal, *Op.* Operation

### Statistik

Die statistischen Analysen erfolgten mit Prism (Version 10, Fa. GraphPad Software, La Jolla, CA, USA). Das Signifikanzniveau wurde auf *p* < 0,05 festgesetzt. Die Normalverteilung wurde grafisch mit Quantile-Quantile-Plots sowie statistisch mit dem D’Agostino-&-Pearson-Test überprüft. Die Ergebnisse wurden, wenn nicht anders angegeben, als Median mit Quartilen sowie als absolute Werte mit Prozenten angegeben. Der Wilcoxon-Test wurde durchgeführt, um Unterschiede zwischen den korrekt identifizierten anatomischen Strukturen vor und nach der Lehreinheit zu untersuchen. Der Mann-Whitney-Test wurde zum Vergleich der Bewertung der Qualitätsmerkmale der 3‑D-Medien und der Anzahl der korrekt identifizierten anatomischen Strukturen nach der Lehreinheit mit einem 3‑D-Medium verwendet.

## Ergebnisse

**Figure 1:**
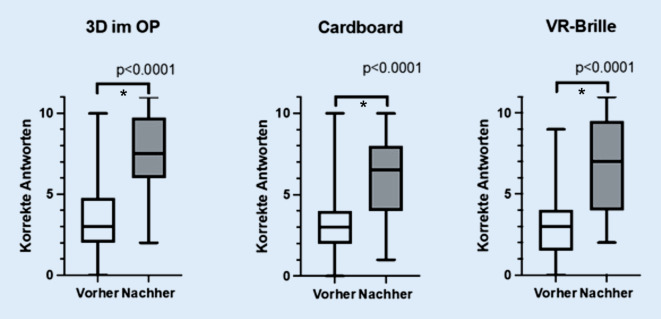

Es nahmen 183 Studierende an der präinterventionellen Wissensstandserhebung teil. Nach Verwendung der 3‑D-Medien beantworteten 91 Studierende vollständig die postinterventionelle Wissensstandserhebung und nahmen an der Evaluation teil. Die Rücklaufquote betrug somit 49,7 %. Es haben 24 Studierende mit einer passiven 3‑D-Brille im OP teilgenommen, 30 die Cardboards verwendet und 37 eine VR-Brille. Die deskriptiven statistischen Ergebnisse der Wissensstandserhebungen sind vergleichend in Tab. 2 dargestellt und in der Abb. 1 visualisiert. Bei der Verwendung der 3 untersuchten 3‑D-Medien wurde ein signifikanter Anstieg der postinterventionell korrekt beantworteten Fragen (*p* < 0,0001; α < 0,05) festgestellt. Der Median erhöhte sich bei der Verwendung der passiven 3‑D-Brillen im OP von 3,0 (Q_1_: 2,0; Q_3_: 4,8) auf 7,5 (Q_1_: 6,0; Q_3_: 9,8), bei den Cardboards von 3,0 (Q_1_: 2,0; Q_3_: 4,0) auf 6,5 (Q_1_: 4,0; Q_3_: 8,0) und bei den VR-Brillen von 3,0 (Q_1_: 1,5; Q_3_: 4,0) auf 7,0 (Q_1_: 4,0; Q_3_: 9,5). Die mediane Differenz betrug im prä- zu postinterventionellen Vergleich bei der Verwendung der passiven 3‑D-Brillen im OP 4,5 und bei den Cardboards und VR-Brillen 3,0.

**Table 2:** 

	OP prä	OP post	CB prä	CB post	VR prä	VR post
Studierende (*n*)	24	24	30	30	37	37
Minimum	0,0	2,0	0,0	1,0	0,0	2,0
25%-Perzentile	2,0	6,0	2,0	4,0	1,5	4,0
Median	3,0	7,5	3,0	6,5	3,0	7,0
75%-Perzentile	4,8	9,8	4,0	8,0	4,0	9,5
Maximum	10,0	11,0	10,0	10,0	9,0	11,0
Spannweite	10,0	9,0	10,0	9,0	9,0	9,0
Mittelwert	3,8	7,5	3,3	6,0	3,2	6,7
SD	2,4	2,5	2,1	2,4	2,3	3,0

*n* = 91; maximal erreichbares Ergebnis: 11 Punkte

Gruppeneinteilung: *OP *3‑D-Gruppe, *CB *Cardboard-Gruppe oder *VR *VR-Brillen-Gruppe

*OP* Operationssaal, *SD *Standardabweichung, *VR* „virtual reality“

**Figure 2:**
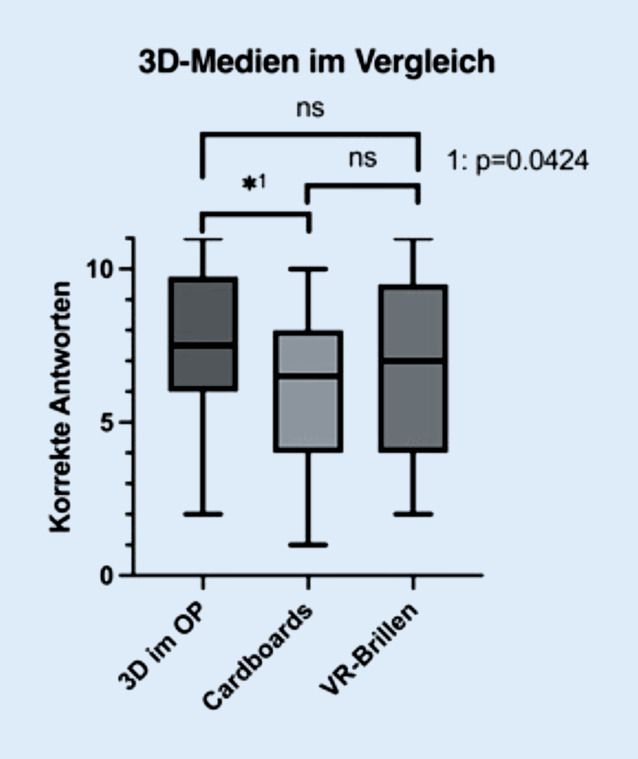

Bei der Betrachtung der postinterventionellen Wissensstandserhebungen (Abb. 2) wurde bei der Verwendung von Cardboards im Vergleich zu 3‑D-Brillen im OP durch die Studierenden signifikant seltener die korrekte Antwort gewählt (*p* = 0,0424; α < 0,05). Im Vergleich zwischen 3‑D- und VR-Brillen sowie zwischen Cardboards und VR-Brillen wurde kein statistisch signifikanter Unterschied in der Leistung festgestellt.

**Figure 3:**
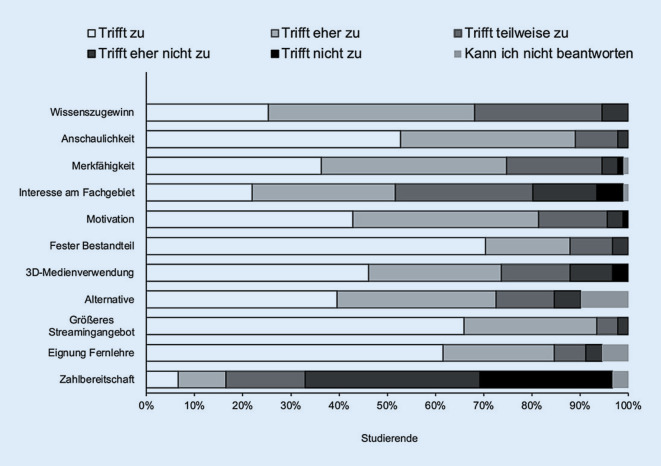

Der Fragebogen der Evaluation ist aus der Tab. 1 zu entnehmen. Die deskriptiven statistischen Ergebnisse sind in Tab. 3 dargestellt und in der Abb. 3 visualisiert. Die Aussagen 1–10 wurden mehrheitlich mit hohen Zustimmungswerten (Median 1,0 und 2,0; „trifft zu“ oder „trifft eher zu“) beantwortet. Lediglich bei der Aussage 11 *„Ich wäre bereit, für das OP-Streaming mit den angewandten Tools Geld zu bezahlen“* wurde mehrheitlich ablehnend (Median 4,0; „trifft eher nicht zu“) votiert. Es wurde kein statistisch signifikanter Unterschied in der Beantwortung zwischen den Untergruppen (3-D-Gruppe, Cardboard-Gruppe oder VR-Brillen) festgestellt.

**Table 3:** 

Aussage	1	2	3	4	5	6	7	8	9	10	11
Studierende (*n*)	91	91	91	91	91	91	91	91	91	91	91
Minimum	1,0	1,0	1,0	1,0	1,0	1,0	1,0	1,0	1,0	1,0	1,0
25%-Perzentile	1,0	1,0	1,0	2,0	1,0	1,0	1,0	1,0	1,0	1,0	3,0
Median	2,0	1,0	2,0	2,0	2,0	1,0	2,0	2,0	1,0	1,0	4,0
75%-Perzentile	3,0	2,0	3,0	3,0	2,0	2,0	3,0	3,0	2,0	2,0	5,0
Maximum	4,0	4,0	6,0	6,0	5,0	4,0	5,0	6,0	4,0	6,0	6,0
Spannweite	3,0	3,0	5,0	5,0	4,0	3,0	4,0	5,0	3,0	5,0	5,0
Mittelwert	2,1	1,6	2,0	2,5	1,8	1,5	2,0	2,2	1,4	1,7	3,8
SD	0,9	0,7	1,0	1,2	0,9	0,8	1,1	1,5	0,7	1,3	1,2

5‑stufige Likert-Skala von 1 („trifft zu“) bis 5 („trifft nicht zu“). 6 entspricht „kann ich nicht beantworten“

*SD* Standardabweichung

**Figure 4:**
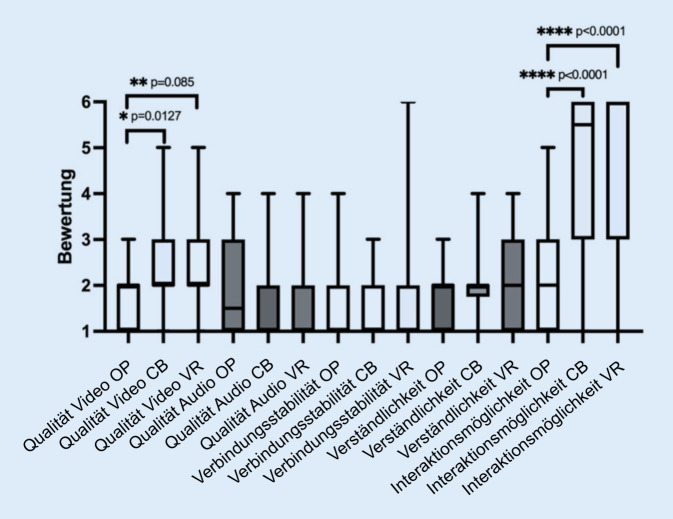

Die deskriptiven statistischen Ergebnisse der Evaluation zu den Qualitätsmerkmalen Video, Audio, Verbindungstabilität zum Stream, Verständlichkeit der intraoperativen Prozesse und Interaktionsmöglichkeit mit dem Operateur sind in der Tab. 4 nach Untergruppen geordnet und in der Abb. 4 dargestellt.

**Table 4:** 

Aussage	1 OP	1 CB	1 VR	2 OP	2 CB	2 VR	3 OP	3 CB	3 VR	4 OP	4 CB	4 VR	5 VR	5 CB	5 OP
Studierende (*n*)	24	30	37	24	30	37	24	30	37	24	30	37	24	30	37
Minimum	1,0	1,0	1,0	1,0	1,0	1,0	1,0	1,0	1,0	1,0	1,0	1,0	1,0	1,0	1,0
25%-Perzentile	1,0	2,0	2,0	1,0	1,0	1,0	1,0	1,0	1,0	1,0	1,8	1,0	1,0	3,0	3,0
Median	2,0	2,0	2,0	1,5	1,0	1,0	1,0	1,0	1,0	2,0	2,0	2,0	2,0	5,5	6,0
75%-Perzentile	2,0	3,0	3,0	3,0	2,0	2,0	2,0	2,0	2,0	2,0	2,0	3,0	3,0	6,0	6,0
Maximum	3,0	5,0	5,0	4,0	4,0	4,0	4,0	3,0	6,0	3,0	4,0	4,0	5,0	6,0	6,0
Spannweite	2,0	4,0	4,0	3,0	3,0	3,0	3,0	2,0	5,0	2,0	3,0	3,0	4,0	5,0	5,0
Mittelwert	1,7	2,3	2,4	1,9	1,5	1,4	1,7	1,5	1,6	1,8	2,0	2,1	2,3	4,5	4,5
SD	0,7	1,0	1,1	1,0	0,8	0,7	0,9	0,6	1,0	0,8	0,7	0,9	1,3	1,7	1,8

Die Bewertung erfolgte entsprechend Schulnoten von 1 („sehr gut“) bis 6 („ungenügend“)

Nummerierung der Aussagen: 1: Video, 2: Audio, 3: Verbindung zum Stream, 4: Verständlichkeit der intraoperativen Prozesse und 5: Interaktion mit dem Operateur

Gruppeneinteilung: *OP *3‑D-Gruppe, *CB *Cardboard-Gruppe oder *VR *VR-Brillen-Gruppe

*OP* Operationssaal, *SD *Standardabweichung, *VR* „virtual reality“

Im Vergleich zeigen sich signifikante Unterschiede bei der Videoqualität zwischen der Verwendung von passiven 3‑D-Brillen im OP und Cardboards (Median 2,0; Q_1_: 1,0; Q_3_: 2,0 vs. Median 2,0; Q_1_: 2,0; Q_3_: 3,0; *p* = 0,0127) sowie den passiven Brillen und VR-Brillen (Median 2,0; Q_1_: 1,0; Q_3_: 2,0 vs. Median 2,0; Q_1_: 2,0; Q_3_: 3,0; *p* = 0,085). Zudem wurde ein signifikanter Unterschied bei der Interaktionsmöglichkeit mit dem Operateur zwischen der Verwendung der passiven 3‑D-Brillen und Cardboards (Median 2,0; Q_1_: 1,0; Q_3_: 3,0 vs. Median 5,5; Q_1_: 3,0; Q_3_: 6,0) sowie zwischen den passiven 3‑D-Brillen und VR-Brillen (Median 2,0; Q_1_: 1,0; Q_3_: 3,0 vs. Median 6,0; Q_1_: 3,0; Q_3_: 6,0) festgestellt. Insgesamt wurde die *Videoqualität* in den Untergruppen mit „gut“ (Median 2,0), die *Audioqualität* mit „sehr gut“ (Median 1,0 und 1,5) die *Verbindungsstabilität* mit „sehr gut“ (Median 1,0), die *Verständlichkeit der intraoperativen Prozesse* mit „gut“ (Median 2,0) und die *Interaktionsmöglichkeit mit dem Operateur* in der Untergruppe 3‑D mit „gut“ (Median 2,0) in den Untergruppen Cardboard und VR-Brillen mit „mangelhaft“ und „ungenügend“ (Median 5,5 und 6,0) bewertet.

## Diskussion

Die digitale Transformation des Gesundheitssystems und der medizinischen Aus- und Weiterbildung ist ein fortlaufender Veränderungsprozess und bezieht sich auf den umfassenden Einsatz von digitalen Technologien und Tools, um das Bildungswesen und den Lernprozess zu verbessern. Neue technologische Innovationen zielen darauf ab, traditionelle Unterrichtsmethoden und Lernumgebungen zu erweitern und zu bereichern, indem digitale Ressourcen, Plattformen und Technologien genutzt werden [15]. Zudem wird durch den Einsatz der elektronischen Lehre ein Wechsel von lehrergelenktem Unterricht zum sog. Flipped-Classroom-Modell beschleunigt. Hierbei steht die digital-virtuelle Lehre nicht mehr in Konkurrenz zur klassischen Präsenzlehre, sondern als sinnvolle Ergänzung. Denn im Sinne der Blended-Learning-Strategie gilt es, kollaborative Tools einzusetzen, um das Lehrangebot und die Zugänglichkeit zu Lerninhalten durch die Verwendung neuer Technologien zu verbessern und zu erweitern [14]. Es muss eine Verbindung zwischen Effektivität und Flexibilität elektronischer Lehrformate mit den sozialen Aspekten der Interaktion zwischen mehreren Personen („face-to-face“) und dem praktischen Erlernen von Tätigkeiten geschaffen werden [8]. Die Entwicklung virtueller Realitäten erlauben durch eine neue Lehrgestaltung verschiedene neue Möglichkeiten der Wissensvermittlung [14]. Gleichzeitig erfordert der Einsatz neuer Technologien eine sorgfältige Planung, Schulung von Lehrpersonal und die Gewährleistung der Datensicherheit und des Datenschutzes.

Die curriculare Lehre der Hals-Nasen-Ohren-Heilkunde an medizinischen Fakultäten ist in Deutschland unterrepräsentiert [16, 17]. Obwohl sie ein interdisziplinär und funktionell bedeutsames Fach ist, werden HNO-ärztliche Themen in der Ausbildung der Studierenden weniger priorisiert, und es werden vergleichsweise wenig Lehrzeiten eingeräumt [18]. Aufgrund der komplexen Kopf-Hals-Anatomie ist die Hals-Nasen-Ohren-Heilkunde enger Partner vieler Fachdisziplinen, um eine ganzheitliche Versorgung von Patienten sicherzustellen. Für die Sicherstellung des Wissenstransfers wird deutlich, dass alternative Lehrmethoden, die zum einen keine zusätzlichen personellen Ressourcen belasten und zum anderen finanzierbar sind, etabliert werden müssen [15].

Die Digitalisierung der studentischen Lehre in den Bereichen der chirurgischen Medizin ist mittlerweile nicht mehr wegzudenken. Sie hat einen hohen Stellenwert, denn die Studierenden als „digital natives“ bewegen sich auf der „Consumer-Ebene“ [4, 19]. Dies bedeutet, dass Menschen, die als „digital natives“ gelten, in erster Linie als Konsumenten digitaler Technologien agieren. Die Consumer-Ebene bezieht sich auf die Rolle als Nutzer und Verbraucher von digitalen Produkten und Dienstleistungen, anstatt auf die Schöpfung oder Produktion solcher Technologien. Die Feststellung, dass „digital natives“ sich auf der Consumer-Ebene bewegen, betont, dass ihre Hauptinteraktion mit Technologie oft auf der Verbraucherseite liegt, indem sie digitale Inhalte konsumieren, mit Apps interagieren und von den Möglichkeiten der digitalen Welt als Endverbraucher profitieren. Dieser Wandlungsprozess und die Nutzung digitaler Technologien auf der Verbraucherebene haben auch einen Einfluss auf die medizinische Lehre und das zukünftige Berufsbild der Ärzte. Der Erfolg kann bisher allerdings nicht abgeschätzt werden. Weder im Nationalen Kompetenzbasierten Lernzielkatalog (NKLM) aus dem Jahr 2015 noch im Masterplan Medizinstudium 2020 werden die Aspekte der digitalen Transformation in die Planung der Lehre detailliert einbezogen bzw. diese adressiert [4, 20, 21]. Bisher ist eine Integration digitaler Lehrmethoden und die Förderung bzw. der Ausbau spezifischer, die digitale Transformation fördernde Strukturen nur eingeschränkt vorhanden. Häufig werden bisher digitale Lehrmethoden, wie Lehrvideos, medizinische Apps oder kollaborative Online-Plattformen für den Wissensaustausch, in Eigenregie durch die Studierenden genutzt [22]. Die COVID-19-Pandemie hat die Notwendigkeit, aber auch die Machbarkeit der Integration digitaler Technologien im Medizinstudium deutlich gemacht. Viele der digitalen Lehrmethoden, die während der Pandemie eingeführt wurden, konnten auch in der Zeit danach erhalten bleiben und in den Lehrplan ergänzend integriert werden [8, 22, 23].

### Vor- und Nachteile der Visualisierungssysteme

Boeker und Klar beschrieben als Voraussetzung für eine erfolgreiche Implementierung von digitaler Lehre und Simulation die allumfängliche und sofortige Integration dieser Formate in die Curricula der Lehreinrichtungen [24]. Betrachtet man dieses Postulat im Kontext der Verwendung von virtueller Realität und 3‑D-Visualisierungssystemen, so zeigt sich eine durch strukturelle Versäumnisse der Vergangenheit entstandene Diskrepanz zwischen den formulierten Zielen der Schaffung einer digitalen Lehrumgebung und den Kapazitäten der Lehreinrichtungen, solche in ausreichend großem Format und Qualität in den Lehralltag frühzeitig zu integrieren [25]. Zwar hat die COVID-19-Pandemie als Katalysator die digitale Transformation vorangetrieben, aber der Rückschritt zum „business as usual“ in der Lehre ist weiterhin sehr verlockend. Daher spielen VR-Simulationen und 3‑D-Systeme zwar zunehmend eine Rolle, jedoch kommen diese aktuell flächendeckend über den Status singulärer Lehrprojekte bisher nicht hinaus [4, 26]. Entscheidungsträger müssen evident von der Notwendigkeit der Verwendung solcher Systeme unter Einhaltung des Prinzips der finanziellen Verhältnismäßigkeit überzeugt werden. Betrachtet man daher die in dieser Studie verwendeten VR-/3-D-Visualisierungssysteme, erscheint die genauere Analyse der Vor- und Nachteile im direkten Vergleich von zentraler Bedeutung. Die Verwendung von 3‑D über passive Brillen im OP kann in dieser Studie als eine statische Methode verstanden werden, da es das Paradigma des Unterrichts in der Klinik unangetastet belässt. Die Studierenden benötigen hierbei keine eigene Hardware, und die Einweisung in die Technologie ist für die Zuschauenden selbsterklärend. Einschränkungen ergeben sich hierbei v. a. durch die personellen Kapazitäten des OP und die vorhandene Menge an 3‑D-Brillen. Zudem muss sichergestellt werden, dass die Zuschauer in einem entsprechenden (limitierten) Betrachtungswinkel den Monitor einsehen können, um eine perfekte 3‑D-Visualisierung zu gewährleisten, was wiederum die Anzahl der Betrachter einschränken kann.

VR-Brillen sowie Cardboards sind im Vergleich mobile Systeme, die für eine große Anzahl an Studierenden örtlich sowie zeitlich flexibel angeboten werden können. Die Möglichkeit der selbstständigen Unterbrechung der Videos und der mehrfachen Wiederholung ist ein großer Vorteil der beiden Gruppen. Aktuelle Studien bestätigen gerade die Wichtigkeit einer erfolgreichen Etablierung von digitalen Lehrmethoden, die sich durch Ungebundenheit in Ort und Zeit charakterisieren [8, 10, 22, 23]. Die Teilnehmenden der Cardboard-Gruppe haben teils von zu Hause der Op. beigewohnt. Es gab allerdings eine Vielzahl der Studierenden an, dass es ihnen nicht möglich war, die gesamte Op. zu verfolgen. Die Visualisierung führte teils zu Müdigkeit, Kopfschmerzen und Übelkeit, sodass die Teilnahme an der Op. unterbrochen oder sogar vollständig beendet werden musste. Dies war bei den qualitativ hochwertigeren VR-Brillen nicht der Fall, allerdings dauerte hier die Einweisung in die Geräte und die Konfiguration der Netzwerkeinstellungen länger. Dies führte häufig, trotz standardisierter schriftlich verfasster Gebrauchsanleitung, zu Rückfragen und damit Bindung von personellen Ressourcen. Studierende, die VR-Brillen trugen, berichteten außerdem von verstärkter Hitzeentwicklung mit Bildung von Kondensat auf den Linsen, die zur Einschränkung der Bildqualität führte. Whang et al. haben die Auswirkung von Hitzeentwicklung auf das subjektive Wohlbefinden bei Verwendung von VR-Brillen untersucht und stellten einen durchschnittlichen Temperaturanstieg von 7,8 °C mit deutlicher Verschlechterung des Bildleistung durch Kondensat bei Verwendung der Brillen über 45 min fest. Hierbei zeigte sich eine Korrelation zwischen dem Temperaturanstieg und der Abnahme des subjektiven Wohlbefindens der Benutzer [27]. Zieht man hingegen eine Studie der Arbeitsgruppe von Schnakenburg et al. hinzu so gaben von 177 Teilnehmern bei Nutzung einer VR-Brille im Kontext eines Mittelohrmodells in der quantitativen Evaluation insgesamt 2 Studierende Kopfschmerzen an, und 10 berichteten von Übelkeit oder Schwindel. Die Nutzdauer betrug hierbei lediglich 15 min [14]. Betrachtet man die Nutzungsdauer von etwa 90 min in der vorliegenden Studie, so sind negative Auswirkungen auf die Performance der VR-Brille/Cardboard und das subjektive Wohlbefinden anzunehmen. Die Bewertungen der qualitativen Merkmale der 3 Visualisierungssysteme in der Evaluation bestätigt diese Annahme. So wird die Videoqualität bei den Cardboards und VR-Brillen signifikant schlechter als bei der Verwendung von 3‑D-Brillen im OP bewertet. Insgesamt müssen daher die Lehreinheiten bei Verwendung dieser mobilen 3‑D-Visualisierungssysteme inhaltlich vollständig und zeitlich so kurz wie möglich konzipiert sein, damit die Vorteile der zeitlich und örtlich unabhängigen Verwendung den Nachteilen einer sinkenden Performance bei längerer Nutzungsdauer überwiegen. Dies erscheint als deutlicher Nachteil dieser beiden Systeme im Vergleich zur OP-Gruppe. Die Qualitätskriterien Audioqualität, Verbindung und Verständlichkeit war bei allen 3‑D-Medien ähnlich. Bezüglich der Wissensstandserhebung zeigte sich ein signifikanter Anstieg der korrekt identifizierten Strukturen von prä- zu postinterventionell bei allen 3‑D-Medien. Auch untereinander zeigten sich keine gravierenden signifikanten Unterschiede postinterventionell. Dies bestätigt, wie bereits auch in verschiedenen vorherigen Untersuchungen in der Klinik der Autoren nachgewiesen, dass sich die Vermittlung von Wissensinhalten für das Fachgebiet der Hals-Nasen-Ohren-Heilkunde aufgrund von exzellenten Visualisierungsmöglichkeiten trotz der genannten Nachteile mit den mobilen VR-Brillen und Cardboards sehr gut bewerkstelligen lassen [8, 10, 14].

Limitierend bei der Umsetzung dieser hier vorgestellten Lehrmethode ist die fehlende Feedback-Funktion in der VR-/Cardboard-Gruppe zu nennen. Es wurde im Live-Stream die Möglichkeit der Studierenden, während der Op. via Chat-Protokoll mit dem Operateur zu kommunizieren, getestet. Jedoch stellte es sich als nicht praktikabel dar, da durch Wechsel aus dem 3‑D-Modus in das Chatfenster die OP-Darstellung unterbrochen wurde. Zudem stellte sich die Schreibfunktion der VR-Brillen mit der Fernbedienung via virtuelle Tastatur als sehr umständlich dar und wurde deshalb nicht verwendet. Dies erklärt die schlechten Ergebnisse bei der Bewertung des Qualitätsmerkmals *Interaktionsmöglichkeit mit dem Operateur*.

### Finanzielle Aspekte

Die Einführung digitaler Lehrmethoden kann sowohl finanzielle Herausforderungen als auch Einsparungspotenziale mit sich bringen. Die Kosten für die Cardboards betragen durchschnittlich zwischen 2 und 5 € und liegen für die VR-Brillen im Bereich von 150–600 €. Finanziell ergaben sich somit allein durch die Anschaffungskosten und ggf. Wartungskosten besonders der VR-Brillen messbare Unterschiede im Vergleich zur „konventionellen“ Lehre. In der Evaluation zeigte sich die Zahlbereitschaft der Studierenden eher gering. Nur 6,6 % der Studierenden wären bereit, für das Streamingangebot der Hals-Nasen-Ohren-Heilkunde mit Cardboards oder VR-Brillen Geld zu zahlen. Es kann vermutet werden, dass bei einer Kohorte mit Studierenden, welche plant, ihre Weiterbildungszeit in der Hals-Nasen-Ohren-Heilkunde zu absolvieren, ähnlich wie auch bei bereits aktiven Weiterbildungsassistenten der Hals-Nasen-Ohren-Heilkunde, die Zahlungsbereitschaft höher ist, da diese höher motiviert sind und ein spezialisierteres Wissen benötigen, um auch chirurgisch-operative Fähigkeiten erlernen zu können [28]. Die langfristigen Vorteile wie erhöhte Flexibilität, verbesserte Lernerfahrungen, Personalentlastung in der Lehre und die Anpassung an moderne Lehrmethoden könnten die anfänglichen Investitionen und laufenden Kosten rechtfertigen.

Zudem ist anzunehmen, dass bei einem potenziellen zukünftigen Einsatz der Technologie im Rahmen der kontinuierlichen medizinischen Weiterbildung (CME) eine erhöhte Zahlungsbereitschaft besteht, da sich diese mit Verdienstausfall bzw. Reisekosten decken würden.

### Technische Herausforderungen

Die Einführung digitaler Lehrmethoden kann technische Herausforderungen mit sich bringen, die es zu bewältigen gilt. So müssen eine funktionierende Hardware und eine stabile bzw. zuverlässige Internetverbindung vorhanden sein, um einen reibungslosen Ablauf zu ermöglichen. Datenschutz und Sicherheit sind bei der digitalen Lehre von höchster Bedeutung. Der unangemessene Umgang mit sensiblen Daten kann zu Problemen führen. Lehrinstitutionen und Lehrende sollten sicherstellen, dass persönliche Informationen der Lernenden, aber v. a. auch Patienten angemessen geschützt werden. Es ist wichtig, die Zustimmung für die Verwendung persönlicher Daten einzuholen und transparent darüber zu sein, welche Daten gesammelt und wie sie verwendet werden. Den Zugang zu digitalen Lernplattformen und -ressourcen gilt es zu kontrollieren, um sicherzustellen, dass nur autorisierte Personen darauf zugreifen können. Bei der Kommunikation zwischen Lehrenden und Studierenden sollten sichere Kanäle verwendet werden, um die Privatsphäre zu schützen. Es sollte eine klare Richtlinie darüber geben, wie lange die gesammelten Daten aufbewahrt werden und wann sie sicher gelöscht werden. Lehrinstitutionen müssen die Datenschutzgesetze und -vorschriften, die in ihrer Region gelten, genau befolgen. Dies kann die Einhaltung von Gesetzen wie der Datenschutz-Grundverordnung (DSGVO) in der Europäischen Union oder anderen regionalen Datenschutzbestimmungen umfassen. Die Bewältigung von technischen Problemen erfordert eine Kombination aus angemessener Schulung, technischer Unterstützung, flexibler Planung und der Auswahl robuster bzw. benutzerfreundlicher Plattformen. Es ist wichtig, dass Bildungseinrichtungen Ressourcen und Strategien bereitstellen, um diese Herausforderungen zu bewältigen und somit eine reibungslose digitale Lehrerfahrung zu gewährleisten [23].

### Limitationen

Laut Hodges et al. sind die Grundprinzipien einer erfolgreichen medizinischen Lehre u. a. gekennzeichnet durch klare Struktur, Feedback-Möglichkeiten und Authentizität [29].

Die fehlende Feedback-Funktion in der VR-/Cardboard-Gruppe wurde bereits als Limitation der 3‑D-Visualisierungsformate genannt. Für die Einhaltung der von Hodges postulierten Prinzipien müssen daher in Zukunft technische Alternativen evaluiert werden. Hierbei wurden bereits erste erfolgreiche Versuche mit einer Spracherkennungssoftware durchgeführt.

Die Methode ist durch die Notwendigkeit einer stabilen Internetverbindung eingeschränkt. Die Erfassung des Lernerfolgs hängt außerdem von den individuellen Kenntnissen des jeweiligen Studierenden ab. Die Identifizierung von anatomischen Landmarken bei der Durchführung einer Cochleaimplantation eignen sich insgesamt sehr gut für eine standardisierte Wissensstandserhebung. Allerdings handelt es sich hier nur sehr bedingt um Wissen, das von Medizinstudierenden erwartet werden kann. Auch wenn die Studierenden Grundzüge im Vorfeld durch die Vorlesungen oder das E‑Learning-Programm übermittelt bekommen haben, ist dies hauptsächlich Wissen für die fachärztliche Weiterbildung und kann als limitierend für diese Studie angesehen werden. Die vorliegende Studie war jedoch eher im Sinne eines methodischen Ansatzes konzipiert.

## Ausblick

Die vorliegende Studie hat gezeigt, dass eine gute Akzeptanz und ein Lernerfolg in allen vorliegenden 3‑D-Medien zu verzeichnen war. Der Einsatz neuartiger Technologien bedingt zudem eine erhöhte Arbeitsentlastung des Lehrpersonals. Allerdings ist es wichtig, sich vor Augen zu halten, dass der Einsatz neuer Technologien, speziell VR-Formate in der Lehre und Chirurgie, noch in der Entwicklungsphase sind und weitere Untersuchungen und Validierungen erforderlich sind, bevor diese neuartigen Technologien weit verbreitet eingesetzt werden können. Derzeit ist der Einsatz neuer Technologien im Rahmen der studentischen Ausbildung noch kein integraler und flächendeckender Bestandteil im Medizinstudium, auch wenn er im Rahmen der curricularen Lehre an Bedeutung gewinnt [4]. Die Nutzung der neuen Technologien kommt bisher nur punktuell zum Einsatz. Sie hat jedoch das Potenzial, den klinischen Alltag und die chirurgische Praxis zu verbessern, indem sie Präzision, Planung, Lehre bzw. Ausbildung und Patientenbeteiligung fördert, da neue Technologien mobile, interaktive und personalisierte Formate darstellen, welche sich an das Lernverhalten der Studierenden anpassen können [4].

## Fazit für die Praxis

- Der Einsatz von Virtual-Reality(VR)-Brillen und Cardboards ermöglicht gleichwertige reale Blicke in den Op.-Alltag.
- Die Ergebnisse zeigen eine große Akzeptanz und eine gesteigerte Motivation bei den Studierenden.
- 3‑D-Visualisierungen bieten in der studentischen Ausbildung zahlreiche Vorteile und werden zunehmend als leistungsstarkes Werkzeug eingesetzt.
- Interaktive digitale Lehrmethoden verbessern und unterstützen bestehende Lehrmethoden und sollten fester und integraler Bestandteil des regulären Lehrplans sein.
- Die vorhandenen technischen Möglichkeiten sind derzeit jedoch nicht geeignet, Präsenzlehre zu ersetzen.
